# Multifunctional Metasurface with PIN Diode Application Featuring Absorption, Polarization Conversion, and Transmission Functions

**DOI:** 10.3390/mi15111344

**Published:** 2024-10-31

**Authors:** Francisco D. M. Nobre, Thayana M. L. de Sousa, Antônio L. P. S. Campos, Maurício W. B. da Silva

**Affiliations:** 1 Department of Electrical Engineering, Federal University of Roraima, Boa Vista 69310-000, CEP, Brazil; nobredie@gmail.com; 2 Telecommunications Engineering Department, Fluminense Federal University, Niterói 24210-201, CEP, Brazil; thayana_mayrink@id.uff.br; 3 Communication Engineering Department, Federal University of Rio Grande do Norte, Natal 59078-900, CEP, Brazil; antonio.campos@ufrn.br

**Keywords:** linear polarization converter, metasurface, absorber

## Abstract

The objective of this paper is to explore the potential of integrating three distinct functionalities into a thin, single-layer metasurface. Specifically, the study introduces a metasurface design that combines absorption, polarization conversion, and transmission capabilities. The proposed structure consists of a double square loop disposed on a dielectric substrate, which is covered by a superstrate. In this study, the traditional ground plane was replaced with a periodic array, selectively reflecting frequencies of interest. Then, the absorption and polarization conversion characteristics were achieved by introducing the resonators in the front layer. By introducing asymmetry to the resonators and integrating PIN diodes for control, we demonstrated that the metasurface could efficiently absorb electromagnetic waves (with PIN diodes in the ON state), convert polarization (with PIN diodes in the OFF state), and enable signal transmission in a different frequency range. The numerical results indicated excellent performance in both absorption and polarization conversion. At a frequency of 3.05 GHz, the absorption rate reached 97%, while a polarization conversion rate of 98% was achieved at the resonance frequency of 4.37 GHz. Moreover, the proposed structure exhibited a thickness of λ/30.7 at the absorption peak.

## 1. Introduction

A metamaterial is a material composed of a mixture of metals and dielectrics. These materials are typically arranged periodically on scales much smaller than the wavelength of the phenomena with which they interact. The properties of metamaterials arise from the characteristics of the substrate, as well as its geometry, size, orientation, and arrangement. When the sizes of the meta-atoms are sufficiently small compared with the wavelength of interest, the macroscopic approach for describing the electromagnetic wave material properties can be applied to metamaterials composed of meta-atoms, similar to materials made of atoms or molecules [1].

Metasurfaces are two-dimensional versions of metamaterials that have become more popular than 3D materials due to their simplicity in the manufacturing process and numerous practical applications [2]. In various applications, metasurfaces can replace metamaterials. Some of their advantages include occupying less physical space than 3D metamaterial structures and forming structures with fewer losses.

Due to their ability to manipulate polarization, which is one of the most important properties of electromagnetic (EM) waves, metasurfaces have enabled new applications in many areas, such as flat lensing, low-profile and low-weight absorbers, beam-switching antennas, and polarization converters [1,2,3,4,5]. Low loss and light weight are attractive features of metasurfaces for applications in diverse frequency ranges of the electromagnetic spectrum [6,7,8,9,10]. However, most metasurfaces provide only a single function per device, which restricts their application to specific frequency ranges. In this sense, the number of researchers interested in developing multifunctional structures using metasurfaces has grown rapidly, opening many new perspectives in different fields of research. As an example, metasurfaces have been efficiently applied to switch between absorption and polarization conversion or to achieve different polarization conversions, such as circular to circular, linear to circular, and linear to cross-linear polarizations [11,12,13,14,15,16]. Many techniques have been developed in addition to designs, both varying according to frequency range and application. Some of the most recent research is presented below.

In [17] the authors propose an active metasurface that can have the functions of absorption and polarization conversion. Switching between different functionalities is achieved by controlling the different states of PIN diodes, which are inserted into the unit cell elements. The proposed structure is dual-band and operates in the Ku band. To achieve multifunctionality, square loops with different dimensions and different dielectric layers were used.

Also, through the implementation of the PIN diode, in [18] the authors propose a multifunctional metasurface for broadband polarization conversion and perfect absorption. The unit cell, with a periodicity of 24 mm, is formed by four layers: the upper layer is composed of a fishbone-like resonator (FLR) combined with a diamond cross resonator (DCR), incorporated by grouped resistors and PIN diodes, etched into the substrate FR-4 dielectric with a thickness of 1.6 mm, and then an air spacer layer with a thickness of 8.5 mm is introduced between the FR-4 substrate and the back composed of FR-4 double-sided copper coated with 0.8 mm thickness. The resonators and metal plate are made of copper with a thickness of 0.035 mm and a conductivity of 5.8 × 10^7^ S/m. The permittivity and loss tangent are 4.3 and 0.02, respectively. The proposed structure operates in the range of 2.97 to 6.03 GHz in polarization conversion mode and in a range covering conversion from 2.56 to 7.62 GHz for absorption mode.

In [19] the authors also designed a configurable metasurface using a PIN diode to obtain the absorption and polarization conversion functions with reasonable results for oblique incidence up to 60°, operating at a frequency of 5.4 GHz for both functions. Its structure, with a periodicity of 16 mm, is composed of a ring and a ring resonator supported by two parallel bars, and the substrate is composed of FR-4, where the permittivity and the loss tangent are 4.2 and 0.025, respectively.

Another technique that can also be used to obtain function integration is structure superposition, used by the authors in [20] for the absorption and polarization conversion functions. The first structure consists of a ¼ circle resonator on two diagonals and the second with a square loop rotated by 45o. Both structures have an FR-4 substrate of 3.2 mm (structure 1) and 0.8 mm (structure 2) with a dielectric constant of 4.3 and a loss tangent of 0.025. The ground plane is composed only of copper. With this configuration, conversion at a frequency of 6.1 GHz and polarization conversion in the frequency band of 7.8–11.9 GHz are obtained. The results demonstrated angular stability of up to 70o for absorption and 50o for polarization conversion.

A metasurface with single-band absorption or dual-band polarization conversion functionalities is proposed in [11]. The different operating states of the proposed structure are achieved by varying the bias voltage applied to varactor diodes and capacitors inserted into the unit cell elements on a flexible substrate. Absorption of 90% is achieved at the 3.9 GHz frequency, while polarization conversion ratios of 96% and 92% are obtained at the 4.6 and 5 GHz frequencies, respectively. The structure has simple geometry, but it is necessary to include different lumped elements in the unit cells. Other examples of metasurfaces that have been efficiently applied for absorption and polarization conversion functions, or even to achieve different polarization conversions, such as from circular to linear and linear to cross-linear, have been developed in [19,21,22,23,24].

To obtain the functional structure, the proposed structures became increasingly robust, in periodicity and thickness, in addition to more complex geometries and increasing the number of resonators. Another important point is that all recently designed structures allow for a maximum of two functions. All these points motivated the creation of the structure presented in this work, a structure with triple function and a single layer.

Therefore, to create structures with simultaneous absorbing and transmitting capabilities, a frequency-selective surface (FSS) in a Dielectric-Metal-Dielectric-Metal (DMDM) composition can be utilized. Breaking the symmetry of these structures results in reduced co-polarization reflection but significantly increased cross-polarization reflection, effectively transforming the structure into a polarization converter.

This work proposes a thin, multifunctional structure capable of serving as an absorber, transmitter, and polarization converter. Multifunctional metasurfaces are crucial for advancing wireless communication and radar technologies. They enable innovative approaches to designing systems that operate in dynamic environments with varying frequency requirements. Another significant advantage is the versatility that metasurfaces offer. Their ability to perform multiple functions within a single structure facilitates integration into various devices and systems, such as antennas, filters, modulators, and sensors.

Additionally, the cost savings and manufacturing simplicity are notable benefits of multifunctional metasurfaces. By consolidating several functions into one structure, these surfaces reduce overall production costs and design complexity. This leads to simpler and more cost-effective manufacturing processes, benefiting both the industry and end consumers with compact, multifunctional devices that require less material and assembly time.

Therefore, the use of multifunctional metasurfaces represents a critical advancement in telecommunications. They provide significant improvements in the efficiency, compactness, performance, and flexibility of systems, with the potential to transform the design and application of communication technologies, radar, and other systems reliant on advanced electromagnetic wave control.

The structure comprises an array of double square loops arranged on a dielectric substrate, with the complete ground plane replaced by a periodic array reflecting only the desired frequencies. When the PIN diode is in the on state, maintaining symmetry, the structure functions as an absorber at 3.05 GHz, achieving an absorption rate greater than 97%, while simultaneously enabling transmission at 4.5 GHz. Thus, with the PIN diode on, the structure can absorb and transmit at two distinct frequencies. Conversely, when the PIN diode is in the off state, the broken symmetry transforms the structure into a polarization converter, reaching a polarization conversion rate of 98% at 4.37 GHz. The design and analysis of absorption, transmission, and polarization conversion were performed using HFSS software, Version 2014.

This paper is organized as follows. Section 2 presents the project to generate the different functions of the structure. Furthermore, the optimal geometric parameters associated with the structure are described. Section 3 presents the comparison of the behavior of the proposed structure with an equivalent RLC circuit, and its parameters are optimized using the ADS Software, Version 2020. Section 4 presents the analyses of the numerical results referring to each functionality through the effective medium theory, as well as the physical mechanisms associated with absorption and polarization conversion. Additionally, the present work compares the designed multifunctional metasurface with recently reported works. Conclusions about the study carried out in this work are made in Section 5.

## 2. Design

**Figure 1:**
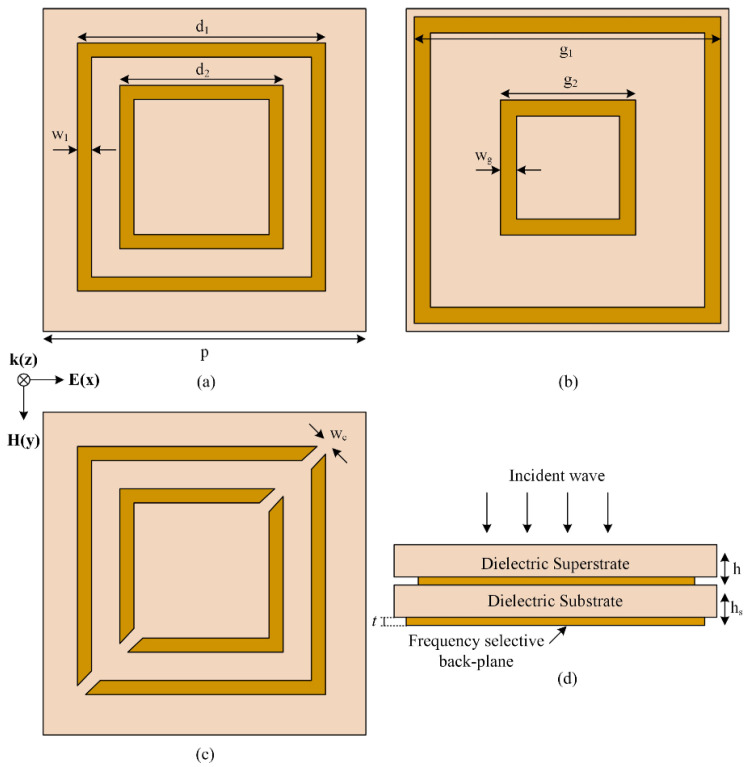
(**a**) Absorber unit cell, (**b**) ground plane view, (**c**) polarization converter unit cell, and (**d**) side view of the structure.

The unit cell of the proposed multifunctional structure is a typical DMDM structure, seen in Figure 1. Figure 1a,c depicts the unit cells comprising the structures in the absorption and polarization conversion configurations, respectively. Figure 1b presents the ground plane view, while Figure 1d illustrates the side view of the proposed structure. To achieve efficient operation, the dielectric thickness was kept much smaller than the wavelength to prevent phase shifts of EM waves and multiple reflections. The proposed structure in this paper used FR-4 as a dielectric layer, with relative permittivity of 4.4 and a loss tangent of 0.02. Additionally, metallic copper with a conductivity σ of 5.8 × 10^7^ S/m and a thickness *t* of 0.035 mm was employed in both layers (back plane and resonators). The geometric dimensions of the structure, as shown in Figure 1, were as follows (all dimensions in mm): d_1_ = 12.4, d_2_ = 8.2, w_1_ = 2, g_1_ = 15, g_2_ = 8.5, and w_g_ = 0.5. The periodicity of the structure was *p* = 17 mm. The side view of the metasurface, seen in Figure 1d, shows that the structure was composed of two dielectric layers (substrate and superstrate) with thicknesses h_1_ = 2.4 mm and h_2_ = 0.8 mm. Both layers were composed of the low-cost FR-4 substrate.

The electromagnetic characteristics, encompassing properties such as complex permeability (μ) and permittivity (ε), can be assessed through either the analytical Drude–Lorentz model [25] or the S-parameter retrieval method [26,27,28,29,30]. The Drude–Lorentz approach exhibits limitations, particularly when applied to metamaterial unit elements of intricate design. Conversely, the S-parameter retrieval method relies on extracting S parameters directly from the physical structure, thereby yielding more precise determinations of permittivity and permeability. These S parameters were derived from HFSS, a robust commercial finite element method (FEM)-based full-wave simulator. The S parameters were used to calculate the refractive index (*n*) and the impedance (*Z*), and finally, these were related to find the values of the effective permittivity (ε*_eff_*) and effective permeability (*μ_eff_*). Through structure simulations, the values were optimized. The effective permittivity (ε*_eff_*) and effective permeability (*μ_eff_*) were related to the refractive index and impedance by the following expressions [31]:

(1) $$\varepsilon_{eff}=\frac{n}{Z}$$

(2) $$\mu_{eff}=nZ$$

where these relationships are dimensionless, provided that the impedance Z is normalized.

**Figure 2:**
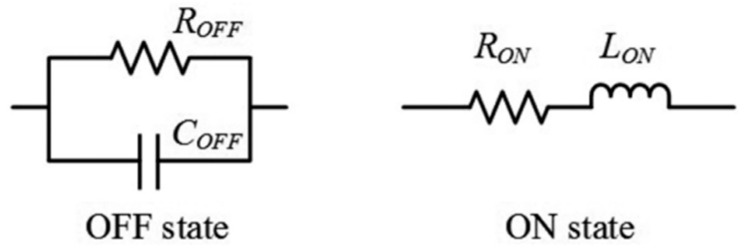
Equivalent circuit of the PIN diode in the off and on states.

In the unit cell was used the BAR64-03W silicon PIN diode, and it was characterized by two distinct states, which were modeled differently. In its off state, it behaved like a parallel circuit with parameters Roff = 3 K Ω and Coff = 0.17 pF. Conversely, in its on state, it acted like a series circuit with parameters Ron = 0.85 Ω and Lon = 1.8 nH. Figure 2 [32] illustrates the equivalent circuit depicting these two states of the PIN diode.

The proposed multifunctional metasurface was composed of simple resonator elements. The unit cell of the proposed structure consisted of a double square loop printed on a substrate, which was covered by a superstrate and backed by a frequency-selective metallic ground plane. When an incident wave hit the structure and the loops were complete, it generated an absorption peak at the design frequency. On the other hand, breaking the symmetry of the elements of the array allowed the structure to offer efficient polarization conversion for the incident wave. In addition, the main objective of this study was to create a structure that allowed absorption and polarization conversion functions while allowing signals from other frequency bands to travel through the structure with minimal or no interference. Therefore, the full ground plane was replaced by a selective ground plane, which was designed to reflect only the frequency bands of interest.

## 3. Equivalent Circuit of the Unit Cell

**Figure 3:**
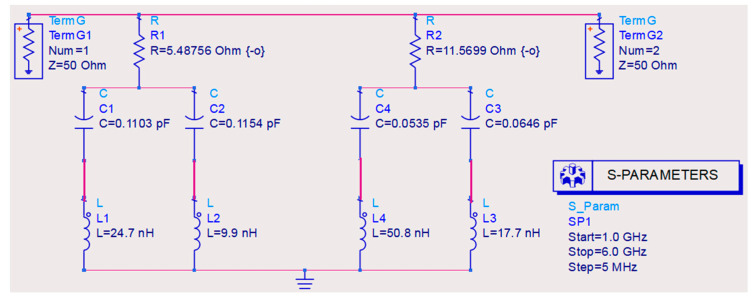
Equivalent circuit of the unit cell.

The unit cell designed and presented in the previous section can be represented by an equivalent electrical circuit, whose main elements are resistors (R), capacitors (C), and inductors (L). Consider that a thin grounded dielectric slab behaves as an inductor if its thickness is less than λ/4 at normal incidence. A single ring-shaped FSS (frequency selective surface) array behaves as a capacitor, so the impedance of a single resonant FSS can be readily modeled with a series LC circuit. In this sense, the number of resonances is related to the number of rings present in the unit cell. Thus, Figure 3 shows the equivalent circuit designed in the ADS Software, where the top part of the unit cell was represented by two parallel LC circuits, composed of elements *L*_1_, *C*_1_, *L*_2_, and *C*_2_, with each LC set representing a ring. Similarly, the ground plane was also represented by two parallel LC circuits, *L*_3_, *C*_3_, *L*_4_, and *C*_4_. Finally, these circuits from the top part and the ground plane were grouped in series, with resistances added to account for losses. The steps to determine this circuit will be presented next [33].

### Determining L and C for Resonance Frequencies

Each resonator of the unit cell (top part and ground plane) has an inductive value, which can be determined by the following equation [34,35,36]:

(3) $$L_{ms}=0.00508L\left[ln\left(\frac{2l}{W+D}\right)+0.5+0.2235\left(\frac{W+D}{l}\right)\right]$$

where $L_{ms}$ is the inductance per unit length of the microstrip (µH), l is the length of the strip (inches) obtained from the dimensions of the coils specified earlier, *W* is the width of the strip (inches), and *D* is the distance between the stripline and the ground plane.

The capacitance values can be obtained using the following equation [37]:

(4) $$C=\frac{1}{4\pi^{2}f^{2}L_{ms}}$$

Considering the case of the absorber, with an operating frequency of 3.05 GHz, and using Equation (3), the following capacitance values were found: *C*_1_ = 0.01103 pF, *C*_2_ = 0.1154 pF, *C*_3_ = 0.0646 pF, and *C*_4_ = 0.0535 pF. Additionally, applying Equation (4), the following inductance values were determined: *L*_1_ = 24.7 nH, *L*_2_ = 9.9 nH, *L*_3_ = 17.7 nH, and *L*_4_ = 50.8 nH. The resistance values were determined using optimization in the ADS software, resulting in *R*_1_ = 5.48756 Ohms and *R*_2_ = 11.5699 Ohms.

**Figure 4:**
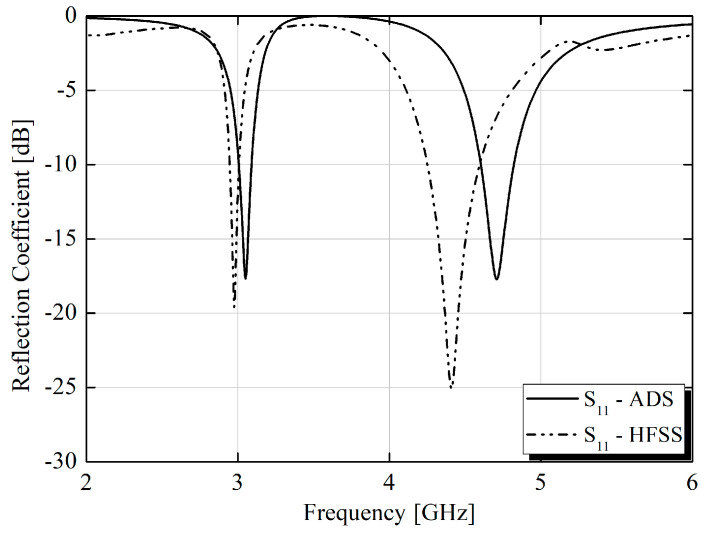
S parameters of the equivalent circuit.

After determining the equivalent circuit, the *S*_11_ parameter values were extracted, and finally, the comparative graph of the *S*_11_ parameters of the structure designed in HFSS and the *S*_11_ of the equivalent circuit, both varying with frequency, is presented in Figure 4. It can be observed that both exhibited similar behavior, particularly at the absorber’s resonance frequency of 3.05 GHz.

It is important to emphasize that, despite the similarity in the behavior of the *S* parameter for the absorber function of the unit cell and the equivalent circuit, a direct comparison between the full electromagnetic wave simulation model and the circuit model was not entirely valid. The circuit model did not account for the generation of reflected waves with both co-polarization and cross-polarization, which limited the accuracy of such a comparison.

## 4. Numerical Results

### 4.1. Absorber Analysis

The first analysis to be performed was whether the proposed absorber should be considered a metamaterial or an FSS, according to the definitions presented in Section 2. Consider the periodicity of the cell *p* = 17 mm, or in terms of the wavelength at the operating frequency (3.05 GHz), 0.1729*λ*. Thus, the periodicity of the absorber was much smaller than the wavelength and met the periodicity requirement of a metamaterial. Furthermore, the thickness of the absorber was 0.0244*λ*, which meant its thickness was also much smaller than the wavelength. Based on these considerations, this absorber could be considered a metasurface, a 2D version of a metamaterial, since its dimensions were subwavelength, unlike an FSS, whose design frequency was directly associated with the electrical length of the structure.

**Figure 5:**
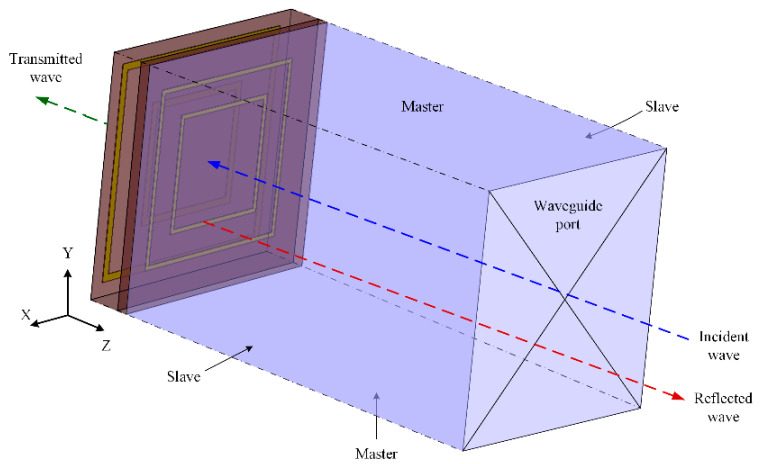
Boundary conditions of the 3D model.

To analyze the behavior of the designed structure, simulations were conducted using the HFSS software environment. The simulation template was adjusted to achieve the desired parameters. After constructing the 3D model of the proposed structure, boundary conditions for the unit cell were incorporated to simulate a periodic arrangement. The boundary conditions, illustrated in Figure 5, included a waveguide port along the *z*-axis for the incidence of the plane wave. Regarding the boundary conditions, two methods were used to achieve periodicity. The combination of perfect electric (PE) and perfect magnetic (PM) boundary conditions simulated periodic boundaries by leveraging the symmetry of the metamaterial through repeated unit cell placement [31]. In addition to these perfect boundary conditions, HFSS provided master–slave boundary conditions to implement periodicity. In this setup, the conditions applied at the master boundary were replicated on the slave boundary, creating an infinitely repeating pattern. While both approaches were effective for cubic unit cells, master–slave boundary conditions also handled complex polygonal structures well [38]. For the unit cell analysis presented, master–slave boundary conditions were employed, as illustrated in Figure 5.

**Figure 6:**
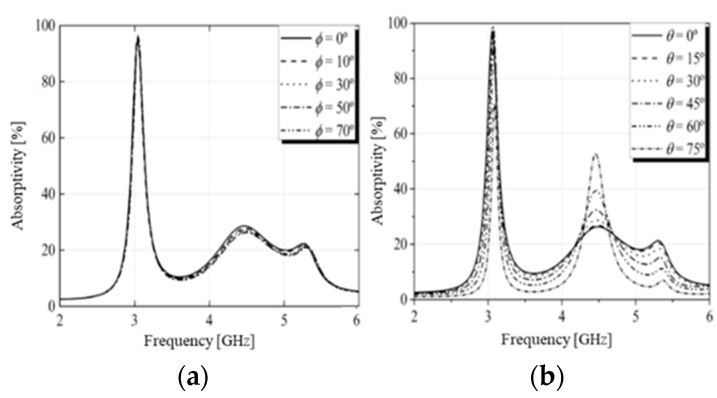
Absorptivity for TE polarization under normal incidence (**a**) and oblique incidence (**b**).

**Figure 7:**
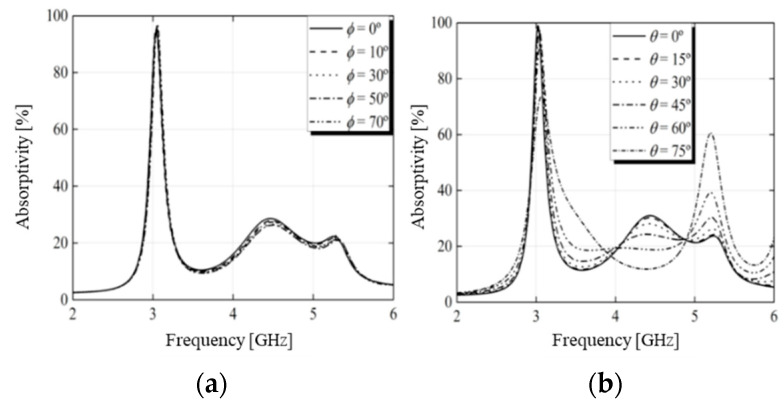
Absorptivity for TM polarization under normal incidence (**a**) and oblique incidence (**b**).

Subsequently, the structure was simulated, and the results of the absorptivity in the absorber configuration for the TE and TM modes are depicted in Figure 6 and Figure 7, respectively. The absorptivity curves for normal incidence of the EM wave and variation of the polarization angle are shown in Figure 6a and Figure 7a. In this study, the E and H fields were rotated in φ. The figures show that there was no change in the absorption coefficient. For oblique angles of incidence, the absorptivity showed little variation, even for large incident angles, indicating that the proposed structure was insensitive to polarization. The absorptivity as a function of the oblique angle of incidence is shown in Figure 6b and Figure 7b. As noted, as the angle of incidence increased, the absorption peak remained almost unchanged for angles of incidence up to 60°, which showed wide-angle stability.

To analyze the absorption performance, the theory of the effective medium [39,40] could be used since the metasurface was a periodic structure of subwavelength. Therefore, the absorption rate could be calculated by the following equations [41]:

*A*(*ω*) = 1 − *R*(*ω*) − *T*(*ω*), (5)

where, *ω* represents the angular frequency, and *R*(*ω*) = |*r_yy_*|^2^ + |*r_xy_*|^2^ and *T*(*ω*) = |*t_yy_*|^2^ + |*t_xy_*|^2^ denote the reflectivity and transmissivity components, respectively. Here, *r_yy_* = *E_yr_*/*E_yi_* and *r_xy_* = *E_xr_/E_yi_* represent the co-polarization and cross-polarization components, respectively. Due to the presence of the lower metallic backplate, *T*(*ω*) tended to approach zero.

**Figure 8:**
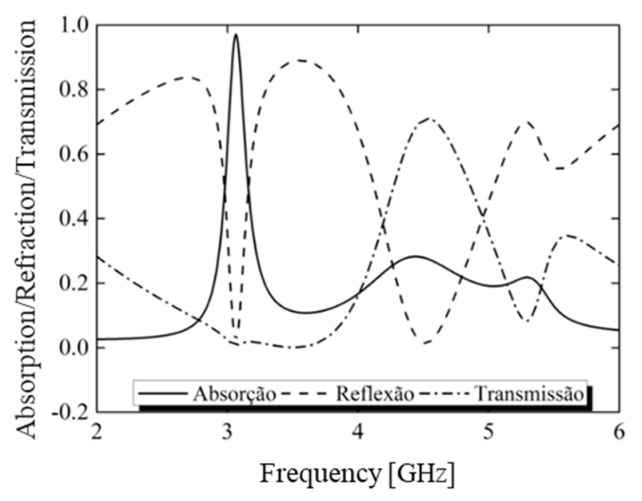
Simulation of reflection, transmission, and absorption of the proposed structure.

The absorption performance of the metasurface is illustrated in Figure 8. It can be observed that *r_yy_* and *r_xy_* caused reflections below −10 dB within the frequency ranges of 3.02 to 3.08 GHz and 4.38 to 4.69 GHz, respectively. While the transmission remained close to zero in the lower frequency range, its value increased at higher frequencies. Consequently, an absorption peak was observed at 3.05 GHz, with an absorption rate of 97%.

**Figure 9:**
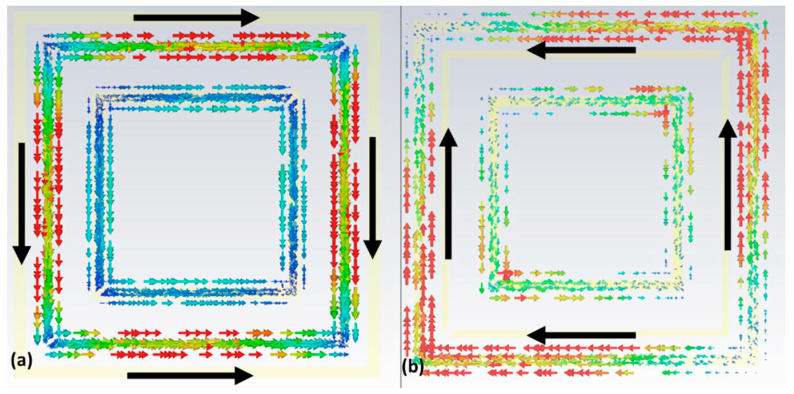
Simulated surface current density for the proposed absorber showing in (**a**) the upper part of the unit cell and (**b**) the ground plane.

To gain a deeper understanding of the absorption mechanism of the proposed metamaterial, surface current distributions at the top and ground plane at the absorption frequency of 3.05 GHz were plotted, as depicted in Figure 9. This graph was essential for confirming the presence of electric and magnetic dipoles, which contributed to the electrical and magnetic resonance responses, respectively. Comparing the top layer with the ground plane, it was evident that the surface current intensity on the ground plane was weaker. This decrease in intensity was due to the attenuation of the incident wave as it propagated through the structure. Additionally, antiparallel currents could be observed, resulting in the circulation of current perpendicular to the applied magnetic field within the structure, thereby exciting a magnetic dipole. Consequently, a magnetic resonance was identified at 3.05 GHz.

While surface current density graphs were effective in indicating the presence of magnetic and/or electrical resonance in the absorber, they alone were insufficient to determine whether the proposed structure was primarily driven by an electric and/or magnetic field. To overcome this limitation, we utilized the single-layer effective medium (SLEM) model, a common approach in the existing literature [41,42,43]. Although our absorber comprised three layers, as detailed in Section 2, employing the SLEM model allowed us to interpret it as a single layer of homogeneous medium. This layer was characterized by its effective relative permittivity (*ϵ*_*eff*_), permeability (*μ*_*eff*_), and normalized input impedance (Z_*eff*_).

**Figure 10:**
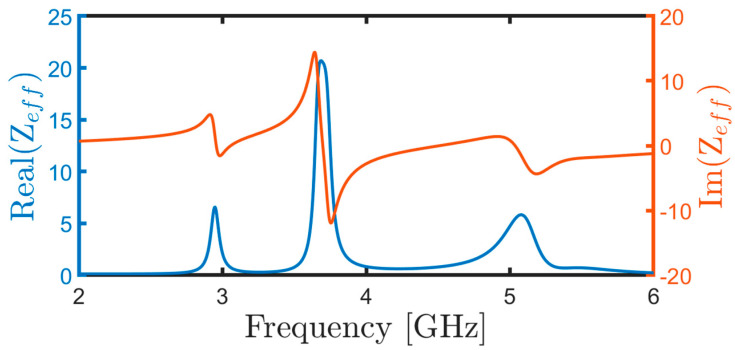
Real and imaginary parts of the normalized input impedance (Z_*eff*_).

Figure 10 illustrates the simulated normalized input impedance Z_*eff*_. Here, the real part of Z_*eff*_ hovered around unity, while the imaginary part approached zero at the absorption peak. This configuration minimized reflection and maximized absorptivity, as described by Equation (3). This behavior stemmed from the reflection coefficient equation, Γ = (Z_*eff*_ − 1)/(Z_*eff*_ + 1).

**Figure 11:**
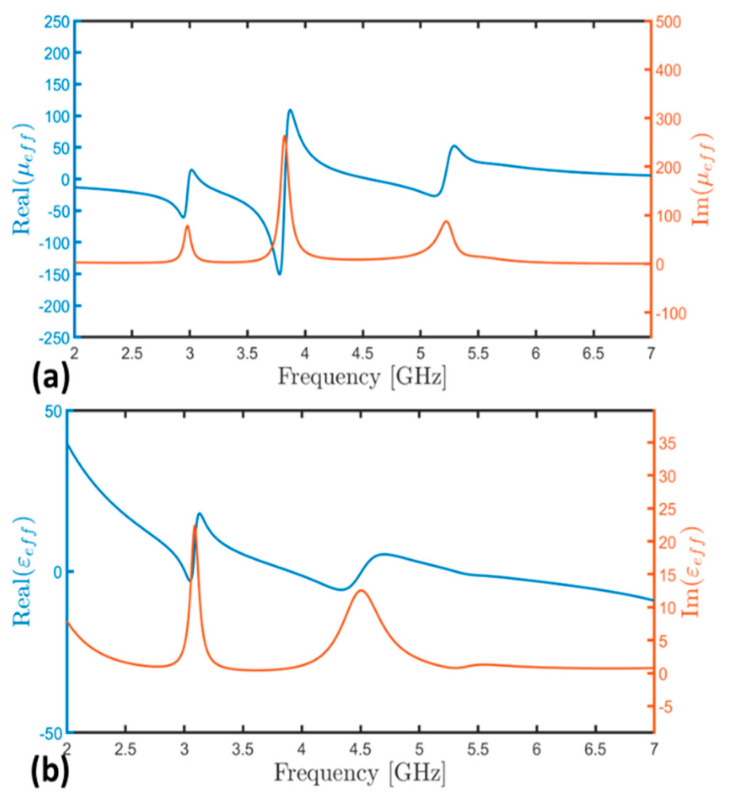
(**a**) Real and imaginary parts of the permeability *μ*_*eff*_ and (**b**) real and imaginary of the effective permittivity *ϵ*_*eff*_.

Moreover, Table 1 provides the real and imaginary parts of the impedance for the metamaterial absorber (MMA) at 3.05 GHz, obtained using the SLEM model. Additionally, Figure 11a,b depicts the real and imaginary parts of *μ*_*eff*_ and *ϵ*_*eff*_, respectively.

In the frequency range from 3.00 GHz to 4.5 GHz, a magnetic response was evident, as indicated by the variation from negative to positive values of the real part of *μ*_*eff*_ (Figure 11a). Conversely, no significant variation was observed in the real part of *ϵ*_*eff*_ (Figure 11b), indicating the absence of an electrical response. This result corroborated the analysis conducted based on surface currents.

**Figure 12:**
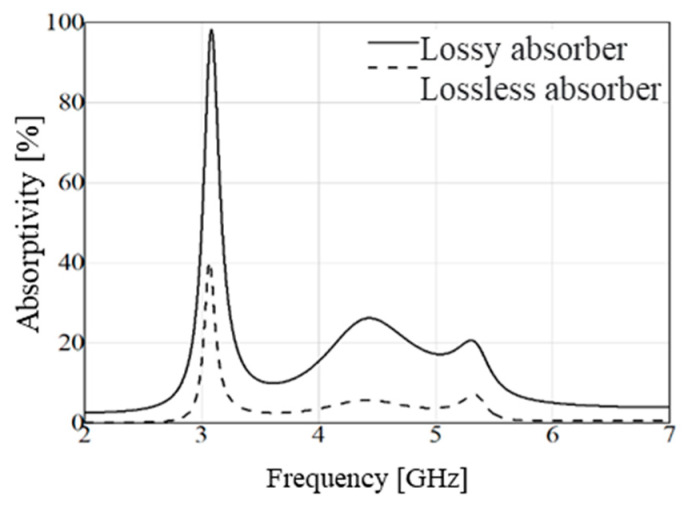
Comparison of absorptivity between an absorbing structure with substrate and superstrate with and without losses.

Understanding the influence of the absorber’s main components on the absorption of the incident electromagnetic wave was crucial. Therefore, the absorptivity of the proposed structure was calculated, considering a substrate and dielectric superstrate without losses. However, it is important to note that a loss tangent of *tanδ* = 0.025 was utilized in the project. The analysis revealed that absorption primarily occurred within the substrate, as evidenced by the low absorptivity level, less than 40%, for the substrate without losses at the main absorption frequency (Figure 12). This underscored the significant role played by the loss tangent of FR-4 in absorption.

**Figure 13:**
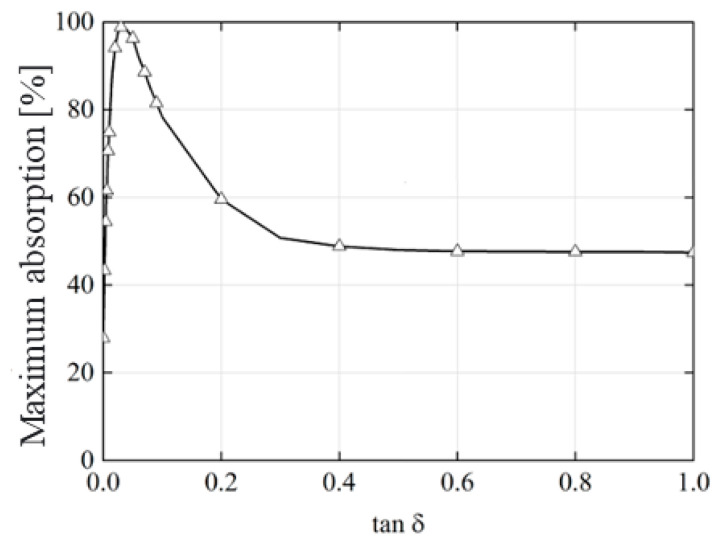
Variation of absorptivity in relation to loss tangent.

As expected, most of the incident wave was absorbed within the substrate. Figure 13 illustrates the performance of the proposed absorber as a function of the substrate loss tangent. It is evident that absorptivity increased rapidly from *tanδ* = 0 to approximately *tanδ* = 0.025, reaching a maximum value of 98%. However, for high loss values, the absorptivity decreased exponentially, dropping below 50%. Thus, it could be concluded that a higher value of *tanδ* did not necessarily lead to greater absorption, as it could affect the input impedance of the absorber, resulting in significant energy reflection.

**Figure 14:**
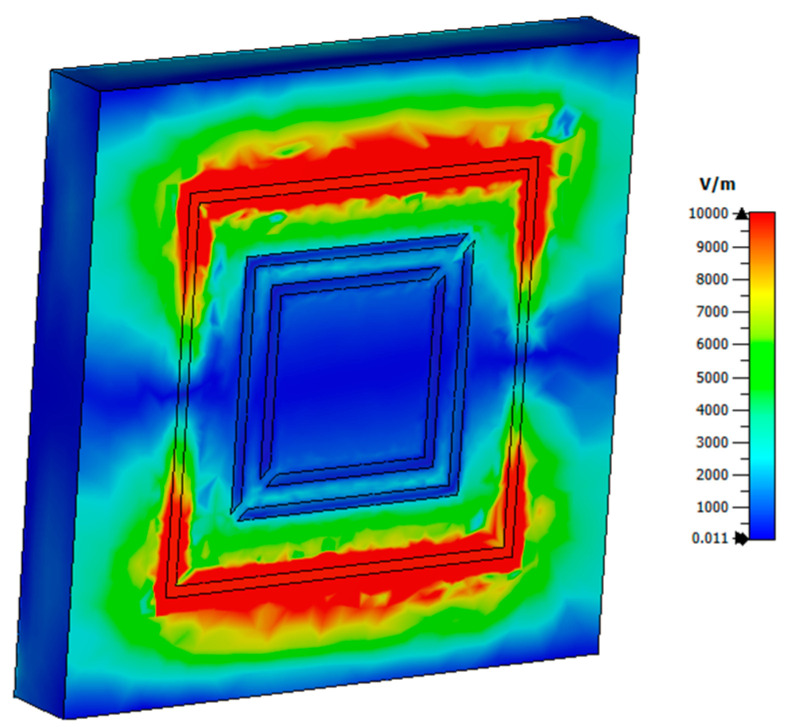
Electric field distribution in the proposed absorber, z-cut.

Figure 14 illustrates the distribution of the electric field in the structure, assuming an electromagnetic wave with normal incidence in the x-direction. The electric field was predominantly concentrated at the edges of the cell, indicating a strong electrical coupling between adjacent elements. This observation suggested the presence of magnetic resonances within the structure.

### 4.2. Polarization Converter Analysis

Thus far, the designed structure has demonstrated capabilities as an absorber and transmitter. However, the objective was to optimize the structure to accommodate additional functionalities. By breaking the symmetry of the resonators, the proposed metasurface could also serve as a polarization converter. When symmetry breaking occurred, the co-polarized reflection component remained low over a wider frequency range, while the cross-polarized reflection component became significantly higher. Consequently, structures with asymmetric resonators exhibited lower absorption levels. The integration of PIN diodes completed the functionality of the structure. When the PIN diode was in the on state, symmetry was maintained, and the structure behaved as an absorber. Conversely, when the PIN diode was in the off state, the symmetry of the array was broken, causing the structure to function as a polarization converter. A detailed analysis of this phenomenon will be presented later.

**Figure 15:**
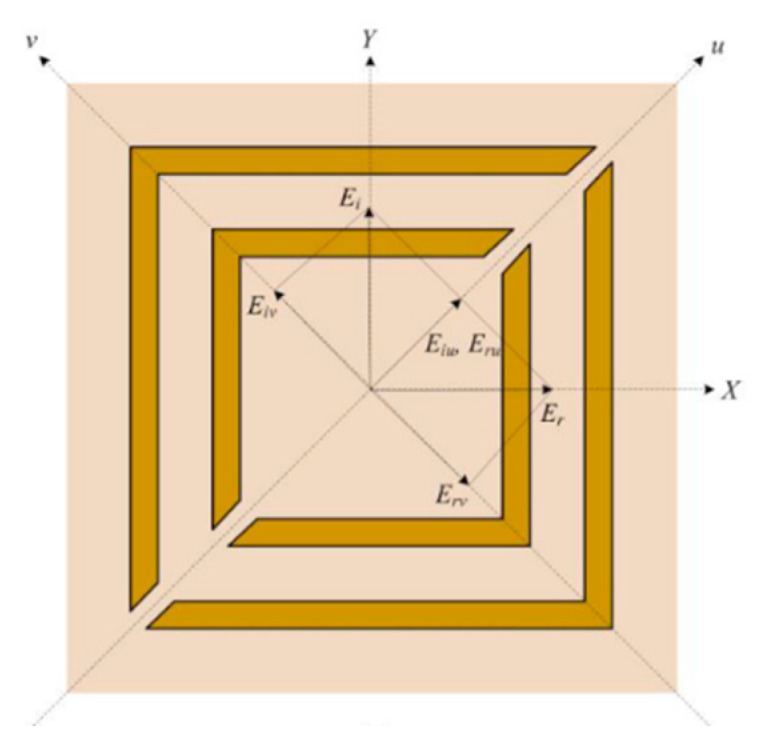
Proposed structure with incident and reflected electric field decomposed into *u* and *v* for conversion of polarization *y* to *x*.

To understand the response of the metasurface conversion and cross-polarization mechanism, the incident electromagnetic wave, polarized along the *y*-axis, could be decomposed into two components, u and v, forming an angle of ±45° relative to the *y*-axis, as depicted in Figure 15.

**Figure 16:**
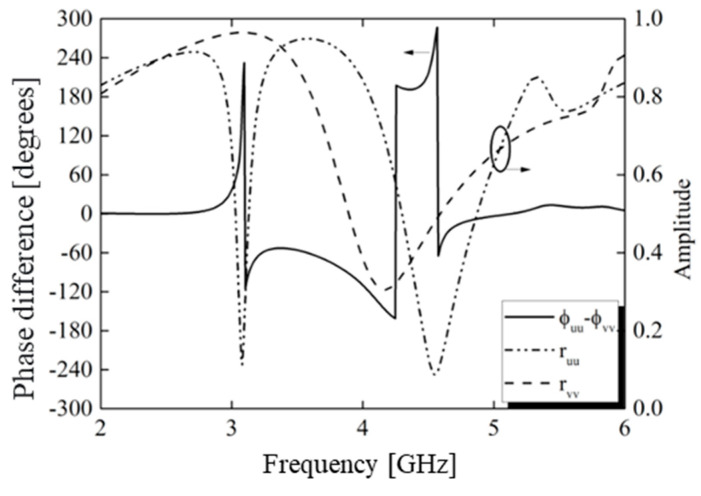
Simulation of reflected amplitudes and phases for incident fields along the *u* and *v* axes.

Numerical simulations of the unit cell were conducted with plane wave incidence, considering polarization along the u and v axes. The results, shown in Figure 16, revealed a phase difference of approximately 180° between the u and v components from 4.24 to 4.45 GHz. This indicated that the metasurface met the conditions for cross-polarization, suggesting efficient polarization conversion within this frequency range.

To demonstrate the efficient polarization conversion capability of the proposed metasurface, we calculated the polarization conversion ratio (*PCR*) for an incident wave polarized along the *y*-axis, defined as [44]

(6) $$PCR=\frac{{\left|r_{xy}\right|}^{2}}{{\left|r_{yy}\right|}^{2}+{\left|r_{xy}\right|}^{2}},$$

The results are shown in Figure 16, where the phase difference between the *u* and *v* components was approximately 180° from 4.24 to 4.45 GHz. In this sense, the metasurface met the cross-polarization conditions, indicating the possibility of polarization conversion in this frequency range. The arrows indicate what axis must be observed for each curve.

**Figure 17:**
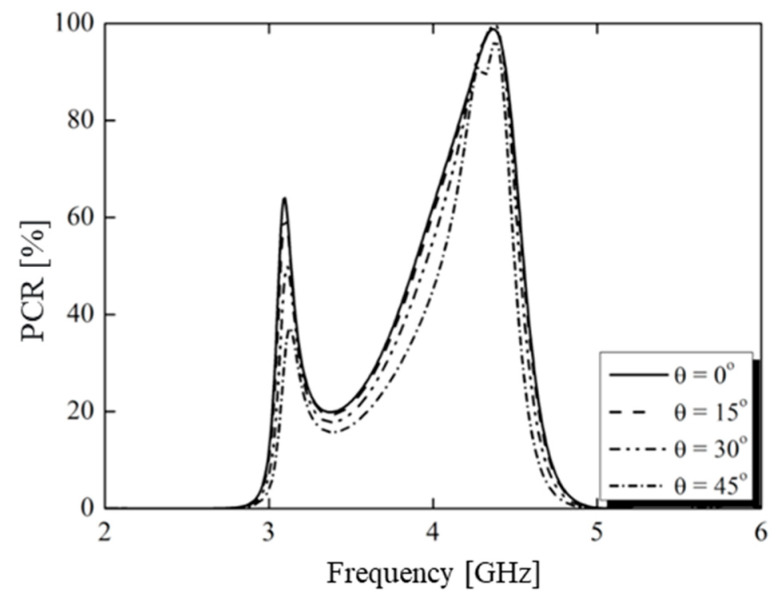
Simulated PCR of the bias converter designed under normal and oblique incidence of EM waves.

As shown in Figure 17, the PCR was greater than 90% in the frequency range 4.24–4.45 GHz, which implied that the linear polarization was properly converted to its cross-polarization waveform and that the device acted as an efficient linear polarization converter. The PCR value at resonance points was 99.8%. We also investigated the PCR for oblique incidence. The obtained results showed that the oblique incidence did not influence the polarization conversion bandwidth.

**Figure 18:**
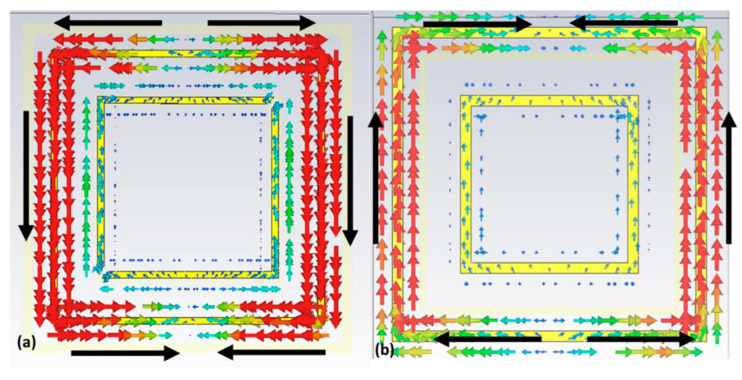
Simulated surface current density for the proposed converter showing (**a**) the unit cell and (**b**) the ground plane.

Also, as analyzed for the absorber, to better understand the conversion mechanism of the proposed metamaterial, the surface current distributions at the top and ground plane at the central conversion frequency of 4.3 GHz were plotted, as can be seen in Figure 18. This graph was important to verify the presence of electric and magnetic dipoles, which provided electrical and magnetic resonance responses. Analyzing the surface currents, it was possible to note that the current densities were antiparallel, and thus, a magnetic resonance was identified at 4.3 GHz.

**Figure 19:**
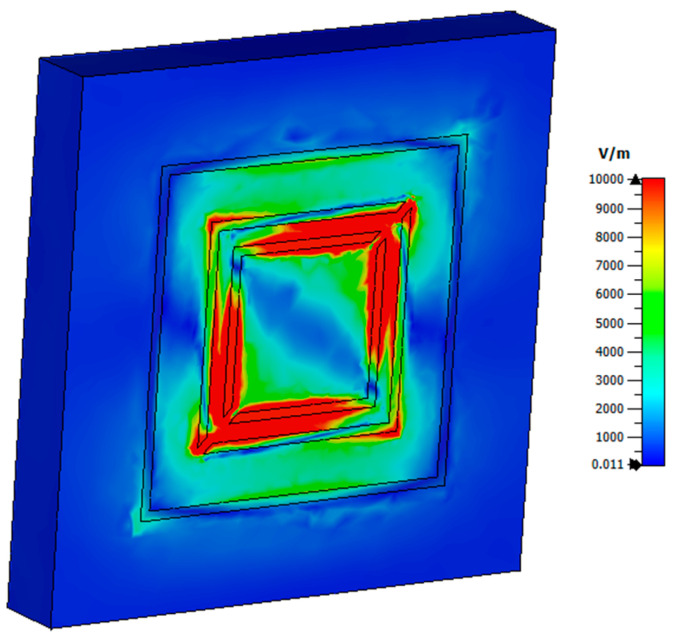
Electric field distribution in the polarization converter proposed, cut in z.

Figure 19 shows the distribution of the electric field in the structure, considering an EM wave with normal incidence in x. The electric field was mainly in the turns and edges where there were no gaps, and a strong electrical coupling between adjacent elements could be observed, indicating the presence of magnetic resonances.

### 4.3. Transmitter Analysis

In conversion mode, the structure also worked by performing transmission, which can be calculated by [44]:

(7) $$T=\sqrt{{\left|t_{xx}\right|}^{2}+{\left|t_{xy}\right|}^{2}}$$

**Figure 20:**
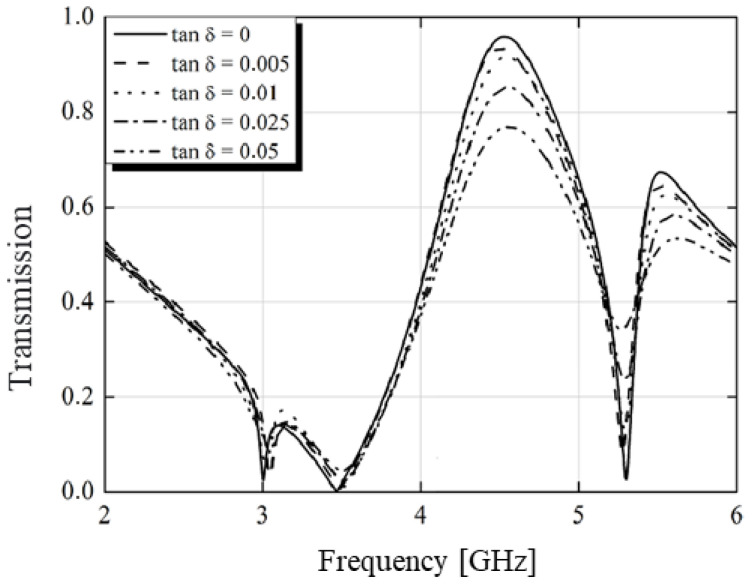
Transmission versus frequency with variation in loss tangent.

**Figure 21:**
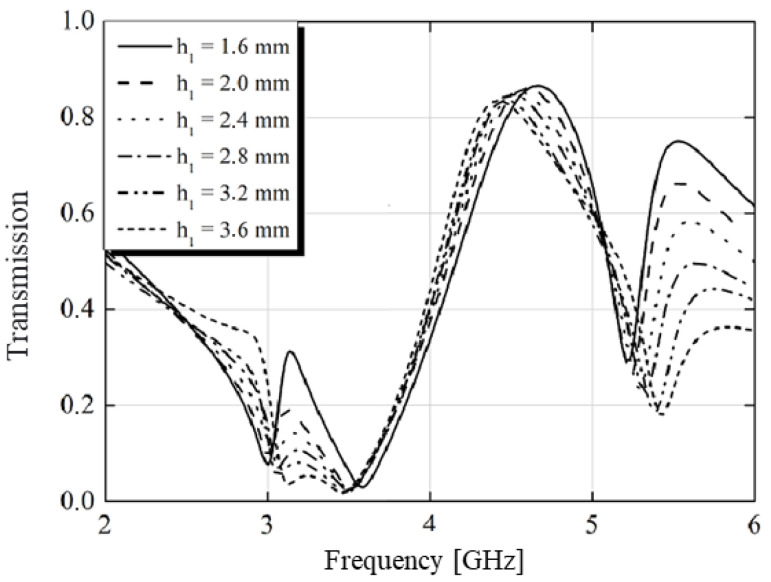
Transmission versus frequency with varying substrate thickness.

By applying Equation (5) and varying the loss tangent and substrate size (h1), we obtained the graphs shown in Figure 20 and Figure 21. The ideal frequency for transmission was 4.5 GHz. It was observed that transmission reached its peak for the case of a lossless structure. However, to fulfill all functions of the structure (including absorption and conversion), a loss tangent of 0.025 was utilized, maintaining transmission above 80%, as depicted in Figure 20. Similarly, when varying the thickness of the substrate, transmission exhibited minimal variations, as illustrated in Figure 21.

### 4.4. Comparison of Results with Previous Work

In Table 2, a series of recent studies related to metasurfaces are presented and were selected due to their functions being similar to those of the structure designed in this work. For the analysis, factors such as functions, number of layers, number of subresonators in the unit cell, absorption frequency, and the relationships between thickness and periodicity with wavelength were considered.

According to the comparison presented in Table 2, a clear improvement could be observed, indicating that the design of this work surpassed previously reported designs by one or more of the mentioned parameters.

## 5. Discussion

This study developed a multifunctional structure capable of functioning as an absorber, transmitter, and polarization converter. The designed structure comprised a series of double square resonators arranged on a dielectric substrate. To selectively reflect only the frequencies of interest, the complete ground plane was replaced by a periodic array, resulting in an absorber configuration. Furthermore, by breaking the symmetry of the structure, it functioned as a polarization converter. The structure design and comprehensive analysis of absorption, transmission, and polarization conversion were conducted using HFSS software. The key features of the structure include its simple design, compact size, and multifunctional operation, enabling absorption, polarization conversion, and transmission across different frequency bands.

The multifunctionality of the structure was achieved through the asymmetry and breaking of symmetry of the resonators, facilitated by the use of a PIN diode. Simulation results demonstrated an absorption rate exceeding 97% at a frequency of 3.05 GHz, a polarization conversion ratio exceeding 98% at 4.37 GHz, and transmission within the frequency range of 4.5 GHz. The absorption function exhibited a bandwidth of 60 MHz, while the polarization conversion function achieved a bandwidth of 210 MHz. Leveraging impedance matching characteristics, ohmic loss at low frequencies, and strong electromagnetic resonance at high frequencies, the proposed structure seamlessly integrated the triple function of absorption and polarization conversion.

As a future direction, efforts will focus on increasing the bandwidth for polarization conversion, as well as constructing and validating a prototype.

## Figures and Tables

**Table 1:** Constitutive electromagnetic parameters of the proposed metasurface absorber.

Frequency (GHz)	Real Part			Imaginary Part		
	*ϵ*_*e* *f* *f*_	*μ*_*e* *f* *f*_	Z_*eff*_	*ϵ*_*e* *f* *f*_	*μ*_*e* *f* *f*_	Z_*eff*_
3.05	0.598	3.473	0.8891	16.71	12.91	−0.0968

**Table 2:** Comparison of results with previous work.

Ref.	Functions	Number of Layers	Number of Resonators in the Unit Cell	Operating Frequencies [GHz]	Cell Thickness/*λ*	Periodicity/*λ*	Technique
[17]	Absorption Conversion	5	2	15.0 17.00	165.25	0.5000	PIN Diode and Layers
[18]	Absorption Conversion	5	2	2.56 to 7.62 2.97 to 6.03	16.4617	0.2048	PIN Diode
[19]	Absorption Conversion	3	4	5.40	85.9489	0.2883	PIN Diode
[20]	Absorption Conversion	5	3	6.10 7.80 to 11.90	83.4689	0.3050	Layers
[11]	Absorption Conversion	3	5	3.90 4.60 and 5.00	26.9180	0.3186	Flexible substrate
[21]	Absorption Conversion	4	3	6.50 to 9.30 12.7 to 17.2	88.256	0.368	Resistor
[22]	Absorption Conversion	4	2	5.80 to 9.40 16.10 to 16.90	78.686	0.1933	Water-based resonator
[19]	Absorption Conversion	3	4	5.40	59.919	0.2883	PIN Diode
[23]	Multiband and multifunctional polarization converter	3	3	15.50 to 16.50 16.00 13.00 18.00	106.950	0.3100	Asymmetric metasurface
[24]	Absorption and cross-polarization conversion	6	2	16.50 to 24.00 4.38 to 11.90	85.702	0.2394	Water-based resonator
This work	Absorption Conversion Transmission	4	4	3.05 4.37 4.50	33.2452	0.1728	PIN Diode

## Data Availability

The original contributions presented in the study are included in the article, further inquiries can be directed to the corresponding author.
